# Italian Immunization Goals: A Political or Scientific Heated Debate?

**DOI:** 10.3389/fphar.2018.00574

**Published:** 2018-05-30

**Authors:** Cristina Scavone, Maurizio Sessa, Emilio Clementi, Francesco Rossi, Annalisa Capuano

**Affiliations:** ^1^Department of Experimental Medicine – Section of Pharmacology “L. Donatelli”, University of Campania “Luigi Vanvitelli”, Naples, Italy; ^2^Department of Drug Design and Pharmacology, University of Copenhagen, Copenhagen, Denmark; ^3^Department of Biomedical and Clinical Sciences, Consiglio Nazionale delle Ricerche Institute of Neuroscience, L. Sacco University Hospital, Università di Milano, Milan, Italy; ^4^Unit of Clinical Pharmacology, Scientific Institute, IRCCS E. Medea, Lecco, Italy

**Keywords:** vaccination coverage, prophylaxis, Law, vaccine hesitancy, Italy

The introduction of vaccinations has represented one of the most important breakthroughs for global health, resulting in significant reductions in mortality and morbidity in the general population with positive impacts also on economic growth (Parretta et al., 2011; Pellegrino et al., 2015; World Health Organization^1^; I vaccini e le vaccinazioni^2^). Still, coverage for highly recommended vaccines is far from optimal, especially in children: 1 in 10 children worldwide did not receive any vaccinations in 2016 with an average of almost 3 million deaths each year due to vaccine-preventable infections (Greenwood, 2014; World Health Organization^3^).

This is not restricted to underdeveloped countries. It has been observed also in developed countries, such as the US, where a decrease in vaccination coverage was observed in children. This was mainly induced by a substantial increase in vaccine hesitancy among parents who chose to excuse their children from school-entry vaccination requirements for non-medical reasons (Delamater et al., 2018). A decrease in vaccination coverage was observed in US also for adult patients, as highlighted by a recent study that analyzed data from the 2015 National Health Interview Survey; it confirmed that, except for a moderate gain for some vaccines, coverage was low (Williams et al., 2017). Likewise, in some European countries vaccination coverage is not adequate (Esposito, 2014). Vaccination coverage is a growing concern in Italy: on December 2017, the Italian Ministry of Health reported 4.885 measles cases and 4 deaths from January 1st 2017. Among those cases, 88% were unvaccinated, while 6% were vaccinated with only one dose^4^. This situation did not escape the attention of the World Health Organization, which underlined how Italy is becoming a problem in the European health scenario, considering that 43% of all measles cases recorded in Europe have occurred in our country. Paradoxically it is the success of vaccination that contributes to the present worsening scenario. The success of vaccination strategies and the almost complete disappearance of some diseases has reduced the perception of the danger of contagion and facilitated the spread of movements opposed to vaccinations, for ethical or religious reasons, or for fear of vaccines-induced adverse events. The negative trend of vaccination in Italy dates back to 2013; this decline involved mandatory vaccinations (anti-diphtheria, anti-polio, anti-tetanic, anti-hepatitis B), and some of those recommended, except for pneumococcal and meningococcal coverage which have instead increased (Pezzotti et al., 2018).

The good news is that Italy has eventually initiated to reverse this course thanks to a new law, dating from last year, the so-called Lorenzin decree, which became law in July 2017 (Law 119/2017 - GU Serie Generale n.182 del 05-08-2017), a move that anticipated the new French law highlighted in a recent Nature Editorial (2018, 2018). The law now renders 10 vaccinations compulsory and free of charge for all pediatric patients aged 0–16. The Italian Ministry of Health have also drawn up a vaccination calendar, which was included in the National Vaccination Prevention Plan (Piano Nazionale Prevenzione Vaccinale) 2017–2019, approved in the State-Regions Conference^5^^,^^6^. In particular, for children from 0 to 6 years, vaccinations which are totally subsidized by the Italian National Health Service now include anti-diphtheria, anti-polio, anti-tetanus, anti-viral hepatitis B, anti-pertussis, anti-*H. influenzae* B, anti-measles, anti-mumps, and anti-rubella (all mandatory for those born in 2001 or later), and anti-chicken pox (mandatory for those born in 2017 or later). Additionally, further vaccinations are strongly recommended in specific patients' age group, such as the anti-meningococcal B and anti-rotavirus for those born in 2017 or later, anti-pneumococcal and anti-meningococcal C for those born in 2012 or later. For teenagers, the booster dose of anti-diphtheria, anti-polio, anti-tetanic, and anti-pertussis are also mandatory for patients born in 2001 or later, anti-HPV for girls and boys (2 times during the 12th year of life), and the tetravalent anti-meningococcal for adolescents. Finally, anti-pneumococcal, anti-zoster and anti-flu for adult over 64 years are recommended^5,6^. Considering its features, the main aims of National Vaccination Prevention Plan are to keep polio-free status, reach the measles- and rubella-free status, ensure the free active offer of vaccinations for frail and vulnerable groups of populations, complete the computerization of the immunological registry, improve the surveillance of preventable diseases by vaccination, and foster research and scientific information on vaccines. The new Law brings novelties also for school registration and attendance. In fact, on March 10th 2018, the deadline for the presentation of compulsory vaccinations' documentation expired. Consequently, approximately 20 children who haven't had all their immunizations were sent home; they will be readmitted as soon as they have been vaccinated.

This new Law is aimed to achieve the highest coverage in immunization, avoiding the return of vaccine-preventable diseases, and to overcome barriers in immunization, including those related to parents and healthcare providers. Recently, the Italian National Institute of Public Health (Istituto Superiore di Sanità) coordinated a survey among parents of children aged 16–36 months, finding that, among 3.130 questionnaires evaluated, vaccine-hesitant parents were 16%, while 39% had doubts about vaccinating their children. According to authors' findings, among the European countries, Italy has the highest levels of skepticism related to effectiveness and safety of vaccines (Giambi et al., 2018). Several factors may have contributed to a decline of confidence in vaccines: the fear of adverse events induced by toxic substances (mercury, aluminum or other dangerous chemical agents); the belief that children's immune system is too immature to respond to vaccines and that the natural exposure to vaccine-preventable diseases provides more enduring immunity; the spread of fake news, especially those related to measles, mumps and rubella (MMR) vaccine-induced autism, despite no evidence existing of a causal association between the MMR vaccine and autism spectrum disorders (Honda et al., 2005; Godlee et al., 2011; Smith, 2017). These results demonstrated that old beliefs, especially those related to the safety profile of vaccines, are still rooted in Italy and that it is neither simple nor immediate to counteract the so-called *vaccine hesitancy* (MacDonald and SAGE Working Group on Vaccine Hesitancy, 2015).

Ten months have passed since the approval of the Law 119/2017, but can we say now that this strategy does work? According to what recently reported by Signorelli et al. (2018), it does; available preliminary data (from June to October 2017) referred to five Italian Regions revealed that, compared with 2016, vaccine coverage rises of 1% for the hexavalent vaccine (against diphtheria, tetanus, pertussis, poliomyelitis, *H. influenzae* B, and hepatitis B) and of 2.9% for the MMR vaccine. Moreover, on March 2018, the Italian National Institute of Public Health declared that the country has reached its goal for hexavalent vaccine coverage (more than 95%)^7^. This increase is expected to bring huge consequences especially for frail and vulnerable population, such as newborn babies, pregnant women, patients without a fully working immune system, including those on chemotherapy treatment and patients affected by HIV. In addition to this, it would be beneficial an increase also in the coverage for flu vaccination that has been falling in European countries in recent years. It would significantly improve clinical conditions of high risk adults and children, elderly and hospitalized patients as well as those affected by chronic cardiovascular, metabolic and respiratory diseases.

The Italian Law, while effective in terms of efficacy in the absence of safety issues, still attracted criticism not only from the anti-vaccine groups but also from politicians especially because of its imperative modalities. We agree with the concept reported in the editorial mentioned (2018), “*Laws are not the only way to boost immunization*”. In our opinion, the continuous monitoring of vaccination coverage, along with the guarantee of free vaccinations to the target population, do represent effective strategies in order to achieve global immunization goals and effectively contrast vaccine concerns not consistent with scientific knowledge. Moreover, an effective risk communication, mainly based on transparency, dialogue, and scientific exchange of information on vaccines between interested parties, may help to solve a worldwide emergency leading to increase in vaccination coverage.

## Author contributions

CS, MS, EC, FR, and AC: Drafting the work and revising it for important intellectual content; CS, MS, EC, FR, and AC: Substantial contributions to the acquisition, analysis, or interpretation of data for the work; CS, MS, EC, FR, and AC: Final approval of the version to be published; CS, MS, EC, FR, and AC: Agreement to be accountable for all aspects of the work in ensuring that questions related to the accuracy or integrity of any part of the work are appropriately investigated and resolved; FR and AC: Developed the concept; CS, MS, EC, FR, and AC: Wrote the paper.

### Conflict of interest statement

The authors declare that the research was conducted in the absence of any commercial or financial relationships that could be construed as a potential conflict of interest.

## Footnotes

^1^World Health Organization. Immunization coverage. Available online at: http://www.who.int/mediacentre/factsheets/fs378/en/ (last access 15-02-2018).

^2^I vaccini e le vaccinazioni. Available online at: https://www.fimp.pro/images/vaccini.pdf (last access 15-02-2018).

^3^World Health Organization. 1 in 10 infants worldwide did not receive any vaccinations in 2016. Available at: http://www.who.int/mediacentre/news/releases/2017/infants-worldwide-vaccinations/en/ (last access 15-02-2018).

^4^Measles in Italy: weekly bullettin. Available online at: http://www.epicentro.iss.it/problemi/morbillo/bollettino/Measles_WeeklyReport_N35eng.pdf (last access 16-02-2018).

^5^Conferenza Permanente per I Rapporti tra lo Stato le Regioni e le Province Autonome di Trento e Bolzano. Available at: http://www.trovanorme.salute.gov.it/norme/dettaglioAtto?id=58185 (last access 15-02-2018).

^6^Aspetti operativi per la piena e uniforme implementazione del nuovo PNPV 2017-2019 e del relativo Calendario Vaccinale. Available online at: http://www.trovanorme.salute.gov.it/norme/renderNormsanPdf?anno=2017&codLeg=58583&parte=1%20&serie=null (last access 15-02-2018).

^7^Vaccine goal achieved, 95% coverage – ISS. Available online at: http://www.ansa.it/english/news/2018/03/12/vaccine-goal-achieved-95-coverage-iss-3_f73aff94-cde5-4430-b1f9-fe89d5248b44.html (last access 12-04-2018).
